# Scholarly concentration programs and medical student research productivity: a systematic review

**DOI:** 10.1007/s40037-017-0328-2

**Published:** 2017-03-27

**Authors:** Annika G. Havnaer, Allison J. Chen, Paul B. Greenberg

**Affiliations:** 1 Program in Liberal Medical Education, Brown University, Providence, RI USA; 2 School of Public Health, Brown University, Providence, RI USA; 3 Division of Ophthalmology, Warren Alpert Medical School of Brown University, Providence, RI USA; 4 Section of Ophthalmology, Providence VA Medical Center, Providence, RI USA

**Keywords:** Scholarly concentration, Scholarly activity, Program evaluation

## Abstract

**Introduction:**

Scholarly concentration programs have become a common method to promote student inquiry and independent research in medical schools. Given the high resource requirements of scholarly concentration program implementation, it is important to examine program efficacy. This systematic review examined the impact of scholarly concentration programs on student research productivity.

**Methods:**

The authors carried out a literature search to find articles related to scholarly concentration program research productivity outcomes. The inclusion criterion was a method of rigorously evaluating program scholarly productivity. Study rigour was evaluated with the Medical Education Research Study Quality Instrument.

**Results:**

The initial search disclosed 2467 unique records: 78 were considered based on titles and abstracts; eight were considered by scanning references. Eleven papers met the inclusion criteria: all were descriptive; none had a priori hypotheses that examined predictors of medical student research productivity in scholarly concentration programs or prospectively evaluated program impact on student scholarly output.

**Discussion:**

While few in number and often lacking in rigour, the studies included herein suggest that adequate administrative support, strong mentorship and tailored program characteristics are essential in facilitating student research productivity in scholarly concentration programs. Given the challenges inherent in medical education research, a conceptual framework based on United Way’s approach may help program planners and educators address this gap in the evaluation of scholarly concentration programs.

**Electronic supplementary material** The online version of this article (doi: 10.1007/s40037-017-0328-2) contains supplementary material, which is available to authorized users.

## What this paper adds

Over the last few decades, scholarly concentration programs have become a common method to promote student inquiry and independent research in medical schools. This systematic review examines the impact of scholarly concentration programs on student research productivity. It underscores the importance of adequate administrative support, strong mentorship and tailored program characteristics in facilitating scholarly output. The review also highlights the potential utility of the United Way model as a conceptual framework for program planners to conduct rigorous scholarly concentration program evaluations.

## Introduction

Medical schools have traditionally utilized a standard approach to medical education, with limited opportunity for scholarship outside the conventional medical curriculum. However, over the past few decades a number of medical schools have implemented scholarly concentration programs to promote student inquiry and independent research [1].

The diverse nature of scholarly concentration programs and the variability in scholarly concentration program descriptors has made it challenging to determine the prevalence of these programs in the United States (US) or elsewhere [1–6]. However, most scholarly concentration programs involve in-depth study beyond the core curriculum, faculty mentorship, a range of concentration areas from which to choose, and a required outcome in the form of a scholarly paper or presentation [1, 2, 4–6]. For example, the scholarly concentration program at the Warren Alpert Medical School of Brown University allows students to undertake a research project in one of thirteen concentration areas [7]: students identify a faculty mentor and area of interest during year one, conduct research during the summer months between years one and two, and continue their research throughout the remaining academic years until they present their scholarly project during year four [7].

A number of factors have prompted schools to implement scholarly concentration programs in recent years. Research experience may confer numerous benefits, including heightened analytical skills, enhanced self-directed learning and knowledge acquisition, improved oral and written communication skills, and the ability to apply new knowledge to patient care [1, 5]. In addition, scholarly concentration programs may help address the shortage of physician-scientists [5]: students exposed to structured research opportunities may be more likely to pursue careers in academic medicine [8]. However, there are potential drawbacks to scholarly concentration programs. In order for students and faculty to participate, a degree of curriculum pruning must occur, and students may devote less time to learning important course material or practising clinical skills [5]. Hence, it is important to consider program goals, potential advantages and opportunity costs prior to scholarly concentration program implementation.

A number of articles have provided detailed descriptions of scholarly concentration programs at various medical schools. Boninger et al. compared the implementation of scholarly concentration programs at two institutions – the University of Pittsburgh School of Medicine, which required participation in a scholarly concentration program, and the Warren Alpert Medical School of Brown University, which offered an elective scholarly concentration program [6]. Though each program offered a broad array of concentration areas (e. g., medical humanities, global health, geriatrics) and required participants to select their own mentor, the scholarly concentration programs differed in several respects. At the University of Pittsburgh School of Medicine, students completed a course series to prepare for the scholarly concentration requirement and received grades at multiple points throughout the program [6]; at the Warren Alpert Medical School of Brown University, scholarly concentration program participants were not required to complete a preparatory course and received no grades [6]. In addition, the University of Pittsburgh School of Medicine funded the costs needed to start and run the scholarly concentration program, which included salary support for administrative staff, information technology personnel for program website maintenance, and compensation for the scholarly concentration directors; at the Warren Alpert Medical School of Brown University, a combination of grant funding, philanthropic support, and funds from the medical school operating budget supported administrative staff and scholarly concentration activities [6]. Finally, while both scholarly concentration programs spanned all four years of medical school, the schools set different deadlines for completing key components (e. g. project proposals).

Medical student participation in scholarly research has grown in conjunction with the implementation of scholarly concentration programs [1, 9]. The Association of American Medical Colleges 2014 Medical School Graduation Questionnaire reported that 69.3% of students conducted a research project with a faculty mentor, a 7.9% increase from 2010 [9]. In addition, 42% of students had sole or joint authorship on a research paper submitted for publication, a 7.4% increase from 2010 [9]. It is unclear if these students were involved in scholarly concentration programs or other research experiences, such as cross-institutional or national initiatives (e. g., research awards from the Doris Duke Clinical Research Fellowship). Given the recent increase in medical student research participation and scholarly output, it is important to critically examine the outcomes of specific research initiatives that promote student research. This is especially true of scholarly concentration programs, which can have high resource requirements and administrative burdens [5].

However, few studies have comprehensively examined the scholarly output of scholarly concentration programs. Chang et al. assessed the benefits of both structured (e. g., mandatory curricular programs, National Institutes of Health-Sponsored Medical Student Research Fellowship programs) and unstructured medical student research activities (e. g., elective summer research, scholarly leaves), and found that the majority of students authored at least one article [10]. However, Chang et al. searched for outcomes associated with *any* type of student research activity, including non-scholarly concentration program research initiatives such as summer assistantships and year-out programs [10]. The distinct features and increased implementation of scholarly concentration programs [1] warrant separate evaluations, however. In their review, Bierer et al. found that the diversity of articles on scholarly concentration programs and variable results precluded definitive conclusions about the value of scholarly concentration programs [1].

In keeping with an evidence-based approach to assessing outcomes in medical education [11, 12], we examined herein student research productivity in scholarly concentration programs and provide a conceptual framework for program planners and educators to conduct scholarly concentration program evaluations. Specifically, we sought to answer the research question: what is the effect of scholarly concentration programs on medical student scholarly output?

## Methods

Given the variability in scholarly concentration program descriptors, we defined scholarly concentration programs as (a) providing an in-depth scholarly experience beyond the conventional curriculum, (b) requiring the completion of a scholarly project, (c) extending for longer than a single summer and (d) occurring primarily at a single institution. We excluded dual-degree tracks (e. g., MD/PhD programs) from our criteria for scholarly concentration programs, as these programs attract students pre-selected for a research career and offer more research opportunities than those available to students in a traditional MD curriculum [13]. In addition, we excluded nationwide initiatives, such as the Howard Hughes Medical Institute Research Fellows Program [14], as these opportunities are available to all medical students and typically involve relocation to other institutions for the project duration. The inclusion criteria were the following: (a) a method of evaluating student research productivity in scholarly concentration programs, such as a presentation, or an abstract or scholarly manuscript accepted for publication and (b) research productivity data for students in either longitudinal cohorts (e. g., scholarly concentration program participants across multiple years) or cross-sectional comparison groups (e. g., scholarly concentration program participants versus non-participants). In addition, we considered only articles written in English. We determined data extraction variables a priori and they were based on the research question.

We carried out a literature search using the databases PubMed, Embase, and Web of Science, and the journals *Academic Medicine, Teaching and Learning in Medicine,* and *Medical Education,* from inception through March 2016, to find articles related to scholarly concentration program research productivity. A health sciences librarian was consulted to formulate search strategies. We searched PubMed using the Medical Subject Headings (MeSH) of ‘students, medical’, ‘schools, medical’, ‘education, medical’, and ‘education, medical, undergraduate’, and keywords including ‘scholarly concentration’, ‘scholarly experience’, ‘scholarly activity’, ‘scholarly program’, ‘research activity’, ‘research experience’, and ‘research product’. We used similar terms to search Embase and Web of Science. Given the variability in database indexing and search platforms, we also searched the journals *Academic Medicine, Teaching and Learning in Medicine, *and *Medical Education* to identify additional articles that our database queries may have missed. See Supplementary file 1 for a full list of search strategies.

**Fig. 1:**
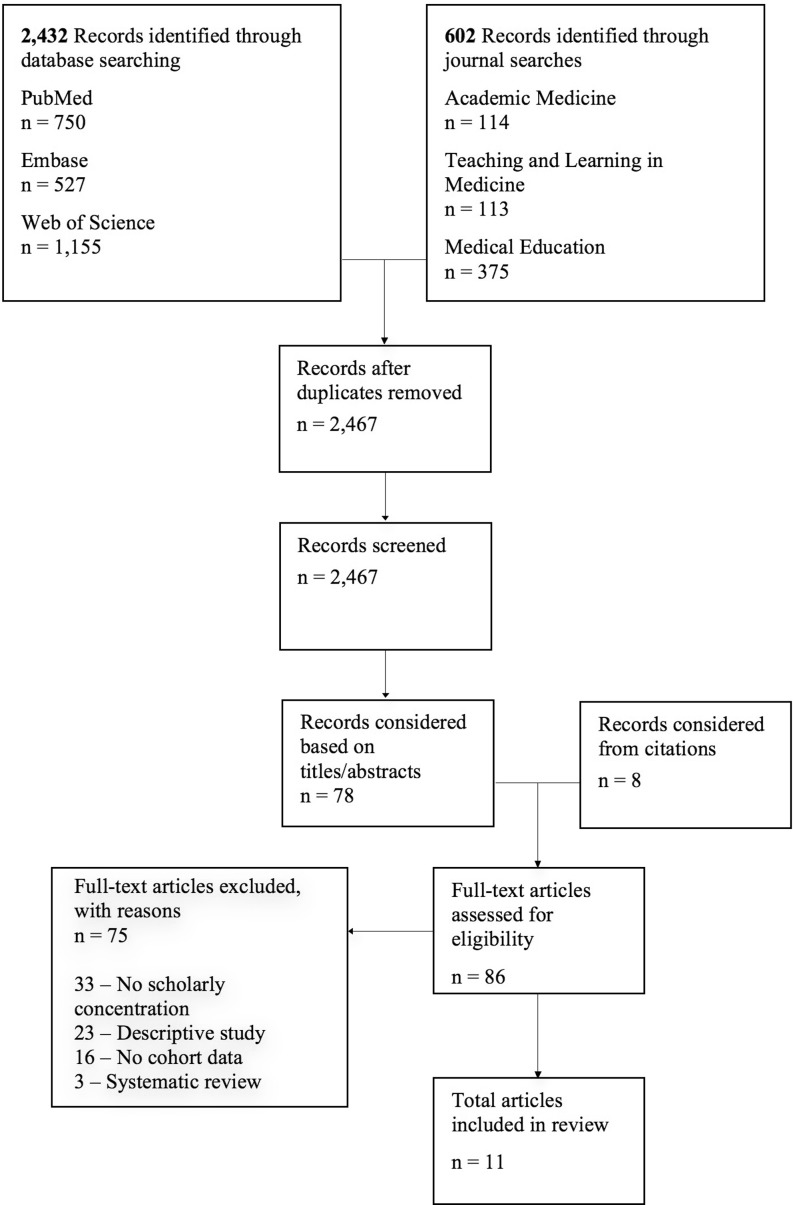
Selection strategy for literature review

Our strategy for article selection is outlined in Fig. 1 and utilized the Preferred Reporting Items and Meta-Analyses checklist and flow diagram [15]. First, we screened articles based on titles and abstracts. Characteristics for article consideration were determined a priori based on pilot searches. We considered an article if the title/abstract mentioned a scholarly concentration program, or if the title/abstract mentioned a medical student research opportunity that could potentially meet our criteria for a scholarly concentration program. Next, the full-texts were read to determine inclusion eligibility. We included articles if they (a) provided data on student research productivity in scholarly concentration programs and (b) provided research productivity data for students in either longitudinal or cross-sectional comparison groups. We excluded articles if they (a) did not discuss a scholarly concentration program, (b) discussed a scholarly concentration program but solely described scholarly concentration program characteristics, (c) provided data on scholarly concentration program research productivity but lacked cohort data for comparison, or (d) provided only secondary data on a scholarly concentration program, such as a literature review. To identify additional papers for consideration, we examined cited references of all articles that discussed a scholarly concentration program, regardless of inclusion in our study. Variables extracted from the articles included institution, research program, country of institution, year published, study design, and student research productivity metrics including both numbers and proportions of student abstracts, publications and presentations. We defined a descriptive study as any study that is not truly experimental [16].

Three independent reviewers (senior medical students) who were otherwise uninvolved in the project evaluated the rigour of the studies using the Medical Education Research Study Quality Instrument [17]. We selected the reviewers based on their significant experience in appraising methodological quality. Prior to evaluating articles, each reviewer was instructed to read two papers that provided detailed descriptions of the Medical Education Research Study Quality Instrument grading system [17, 18]. We used the Medical Education Research Study Quality Instrument as it is specifically designed to evaluate methodological quality of medical education research [17]. If a study utilized multiple methodologies, the highest possible score for each Medical Education Research Study Quality Instrument item was recorded. Reviewer scores were averaged for each of the six domains of study quality: study design, sampling, type of data, validity, data analysis, and outcomes. The maximum possible score for each domain was 3. In addition, the average total score for each study was calculated as the percentage of total achievable points and then adjusted to a standard denominator of 18 to account for ‘not applicable’ responses. As per the recommendations set forth by Cook et al. [18], we focused our interpretations on item-specific rather than overall scores, and used median normative scores as reference points rather than absolute indicators of high and low quality thresholds. To gauge the reliability of the ratings, inter-rater reliability was calculated for each item and total scores using the *icc* package in STATA 14 (StataCorp. 2015. Statistical Software. College Station, TX). Thresholds set forth by Landis and Koch were used to classify inter-rater reliability (0.21–0.4 = fair, 0.41–0.6 = moderate, 0.61–0.8 = substantial, and 0.81–1 = almost perfect) [19].

## Results

Our initial search disclosed 2467 unique records; 78 were considered based on titles and abstracts and eight were considered by scanning cited references (Fig. 1). Eleven papers met our inclusion criteria (Table 1); of these, one was retrieved from a journal search [22]. All were primarily descriptive. Eight studies were retrospective. In general, the studies found that scholarly output increased with scholarly concentration program implementation [20, 22, 23]. Gonzales et al. reported that the number of student presentations resulting from a family medicine scholarly concentration program increased from zero to seven between one and seven years after program implementation [20]. Similarly, Ogunyemi et al. reported that presentations resulting from a primary care scholarly concentration program increased from five to ten, which was attributed to an increased emphasis on presentation at professional conferences [22].

**Table 1:** Selected studies on the impact of scholarly concentration programs on medical student research productivity

Study; year published	Institution; name of scholarly concentration	Country	Methodology	Average Adjusted MERSQI Score	Research outcomes
Elwood et al. 1986 [26]	Nottingham Medical School; Honours Year Program in Community Health	UK	Survey (*n* = 98; 80% response rate)	7.8	– The proportion of publications and presentations resulting from a required research program increased from 13% to 53% from the first through fifth and tenth through thirteenth graduating classes following program implementation.
Gonzales et al. 1998 [20]	University of Colorado; Family Medicine Scholars Program	US	Retrospective^a^ (*n* = 161)	9.3	– The number of student presentations resulting from a family medicine research program increased from zero to seven between one and seven years following program implementation. – The number of papers accepted for publication increased from zero to two over the same time period.
Smith et al. 2001 [27]	University of Calgary Faculty of Medicine; Research Project Program	Canada	Survey (*n* = 63; 91% response rate); compared to survey data collected 10 years earlier	9.7	– The proportion of students who had submitted or were planning to submit their research for publication increased from 11% to 59% between the first and tenth graduating classes, respectively, following implementation of a mandatory scholarly concentration program.
Solomon et al. 2003 [8]	University of Tennessee College of Medicine; Medical Student Research Fellowship	US	Survey (*n* = 114; response rate not specified)	11.2	– The number of abstracts and presentations resulting from a medical student research fellowship increased from 7 to 60, and 6 to 52, respectively, from the 17^th^ to 21^st^ years following program implementation. – The number of manuscripts decreased from 14 to 13 over the same time period.
Ogunyemi et al. 2005 [22]	Drew University of Medicine; Mandatory Medical Thesis	US	Retrospective^a^ (*n* = 168)	9.0	– The number of presentations resulting from a mandatory primary care research thesis increased from five to ten between seven and eight years following program implementation.
Zier et al. 2006 [13]	Mount Sinai School of Medicine; Medical Student Research Program	US	Retrospective (‘*n*’ not provided)	10.5	– The percentage of graduating students publishing peer-reviewed manuscripts increased from 11 to 25% between two and eight years following implementation of structured research programs. – The percentage of students as first authors increased from 5 to 13% within the same period.
Dyrbye et al. 2008 [23]	Mayo Medical School; Required Third-Year Medical School Research Experience	US	Retrospective cohort (*n* = 981)	12.9	– Graduates who published a research report *related* to their required scholarly concentration – relative to their peers who did not – published more research reports *unrelated* to their required research within three years of graduation. – Graduates who presented their scholarly concentration research or published an abstract *related* to their scholarly concentration published more *total* and *unrelated* research reports within three years of graduation. – More students in a required 21-week research experience were first authors than those in a 17/18-week experience.
Langhammer et al. 2009 [29]	Robert Wood Johnson Medical School; Distinction in Research Program	US	Retrospective^a^ (*n* = 28)	14.3	– The proportion of students who submitted a published manuscript to fulfil an elective research program final product requirement was 1/1 (100%), 1/6 (17%), 1/9 (11%), 2/4 (50%), and 0/8 (0%) for academic years 2003, 2004, 2005, 2006, and 2007, respectively.
Akman et al. 2010 [21]	Marmara University School of Medicine	Turkey	Retrospective (*n* = 289)	12.5	– The number of poster presentations by first year medical students in a mandatory group research program was 11, 17, 23, 38, 22, and 32 for years 2002 through 2007, respectively. – The number of oral presentations by second and third year participants in the same program was 18, 25, 31, 26, 36, and 38 for years 2002 through 2007, respectively; the number of poster presentations by second and third year participants was 19, 19, 37, 31, 19, and 25 over the same time period.
Areephanthu et al. 2015 [24]	Professional Mentored Student Research Fellowship; University of Kentucky College of Medicine	US	Retrospective (119 research program participants versus 898 non-participants)	13.8	– Students enrolled in an elective research program authored 0.8 ± 0.3 papers, versus their classmates who were not enrolled (0.3 ± 0.06, *p* < 0.0001). – Research program participants were more than twice as likely than their classmates to author or co-author a PubMed-indexed paper (36.7% vs. 17.9%, *p* < 0.0001). – Of students that published PubMed-indexed papers, research program participants published 2.1 ± 0.51 publications compared to 1.4 ± 0.15 for non-participants (*p* < 0.001).
George et al. 2015 [25]	Scholarly Concentration Program; The Warren Alpert Medical School of Brown University	US	Retrospective (*n* = 460)	14.1	– Participants in an elective research program published an average of 1.29 papers per student, compared to 0.83 papers published per non-participant. – Program participants had a higher frequency of publications than non-participants; the difference was statistically significant (*p* = 0.013).

*MERSQI* Medical Education Research Study Quality Instrument

^a^Not specified whether data collected retrospectively or prospectively

Two studies found a statistically significant difference in publications between students enrolled in an elective scholarly concentration program versus students who were not enrolled [24, 25]: Areephanthu et al. reported that scholarly concentration program participants authored a mean of 0.8 publications compared with 0.3 for their peers [24]; similarly, George et al. reported that scholarly concentration program participants published a mean of 1.3 papers compared with 0.8 papers for their classmates [25].

Three studies surveyed scholarly concentration program participants: Elwood et al. reported that publications and presentations increased from 13 to 53% [26]; Solomon et al. found that the number of abstracts and presentations increased more than eight-fold [8]; and Smith et al. reported that the proportion of students who had submitted or were planning to submit their research for publication increased from 11 to 59% [27].

The average Medical Education Research Study Quality Instrument scores for each of the six domains of study quality were as follows: study design (1.5), sampling (1.6), data type (2.8), study validity (1.2), data analysis (2.5), and outcome (1.5) (Table 2). The average total adjusted Medical Education Research Study Quality Instrument score for all studies was 11.4 out of 18 possible points. Inter-rater reliability for the total Medical Education Research Study Quality Instrument scores was 0.92, and ranged from near zero to 1 for individual items (Table 3).

**Table 2:** Comparison of individual reviewer (A, B, C) Medical Education Research Study Quality Instrument scores for studies included in review

	Overall scores	Domain-specific scores^a^											
*First author (year)*	*Average adjusted total* ^b^	*Study design*				*Sampling*				*Data type*			
		A	B	C	Mean	A	B	C	Mean	A	B	C	Mean
Elwood (1986) [26]	7.8	2	1	1	1.3	2	2	2	2	1	1	1	1
Gonzales (1998) [20]	9.3	1	1	1	1	0.5	0.5	1	0.7	3	3	3	3
Smith (2001) [27]	9.7	2	1	1	1.3	2	2	2	2	3	3	3	3
Solomon (2003) [8]	11.2	2	1.5	2	1.8	2.5	2.5	2.5	2.5	3	3	3	3
Ogunyemi (2005) [22]	9.0	1	1	1	1	1	2	2	1.7	3	3	3	3
Zier (2006) [13]	10.5	1	1	1	1	2	1	1.5	1.5	3	3	3	3
Dyrbye (2008) [23]	12.9	1	2	2	1.7	2	2	2	2	3	3	3	3
Langhammer (2009) [29]	14.3	2	2	2	2	2	0.5	0.5	1	3	3	3	3
Akman (2010) [21]	12.5	1	1.5	1.5	1.3	1.5	2	2	1.8	3	3	3	3
Areephanthu (2015) [24]	13.8	2	2	2	2	2	0.5	0.5	1	3	3	3	3
George (2015) [25]	14.1	2	2	2	2	2	0.5	0.5	1	3	3	3	3
Total/Average	11.4	–	–	–	1.5	–	–	–	1.6	–	–	–	2.8
*First author (year)*		*Validity*				*Analysis*				*Outcome*			
		A	B	C	Mean	A	B	C	Mean	A	B	C	Mean
Elwood (1986) [26]	–	0	N/A	0	0	2	2	2	2	1	1	1	1
Gonzales (1998) [20]	–	N/A	0	N/A	0	1	2	2	1.7	2	1	1	1.3
Smith (2001) ([27])	–	0	0	0	0	2	2	2	2	2	1	1	1.3
Solomon (2003) [8]	–	2	0	0	0.7	2	2	2	2	2	1	1	1.3
Ogunyemi (2005) [22]	–	0	0	0	0	2	2	2	2	2	1	1	1.3
Zier (2006) [13]	–	3	0	0	1	2	3	3	2.7	2	1	1	1.3
Dyrbye (2008) [23]	–	2	2	1	1.7	3	3	3	3	2	1	1	1.3
Langhammer (2009) [29]	–	3	N/A	N/A	3	3	3	3	3	2	2	2	2
Akman (2010) [21]	–	1	2	1	1.3	3	3	3	3	2	2	2	2
Areephanthu (2015) [24]	–	2	N/A	N/A	2	3	3	3	3	2	2	1.5	1.8
George (2015) [25]	–	3	N/A	N/A	3	3	3	3	3	2	1.5	2	1.8
Total/Average	–	–	–	–	1.2	–	–	–	2.5	–	–	–	1.5

*N/A* not applicable

^a^Maximum possible score for each domain is 3

^b^Adjusted to a standard denominator of 18 to account for ‘not applicable’ responses

**Table 3:** Intraclass correlation coefficient values for individual Medical Education Research Study Quality Instrument item scores and adjusted total Medical Education Research Study Quality Instrument scores for studies included in the review

Medical Education Research Study Quality Instrument item	Intraclass correlation coefficient (95% CI)	Fraction of papers with 100% agreement among raters^a^
Study design	0.79 (0.41, 0.94)	6/11
Sampling		
Number of institutions	1.00 (1.00, 1.00)	11/11
Response rate	0.16 (−3.09, 0.85)	4/7
Type of data	1.00 (1.00, 1.00)	11/11
Validity of evaluation instrument		
Internal structure	–^b^	3/5
Content	0.83 (0.37, 0.97)	4/6
Relationships to other variables	–^b^	3/6
Data analysis		
Appropriateness of analysis	–^b^	10/11
Complexity of analysis	0.96 (0.89, 0.99)	10/11
Outcomes	0.56 (−0.01, 0.86)	3/11
Adjusted total^c^	0.92 (0.74, 0.98)	–

*CI* confidence interval

^a^Medical Education Research Study Quality Instrument item scores for some papers were omitted from the computations if they received a score of “not applicable”; hence, the number of papers included in the denominator is variable among items

^b^Intraclass correlation coefficient estimates were not applied to items for which the range of scores between papers was limited

^c^Adjusted to a standard denominator of 18 to account for ‘not applicable’ responses

## Discussion

A scholarly concentration program is a promising initiative in undergraduate medical education but this systematic review underscores the dearth of evidence supporting the efficacy of scholarly concentration programs in promoting medical student research productivity. In general, the studies included herein suggest that adequate administrative support, strong mentorship, and tailored program characteristics are essential in facilitating scholarly concentration program output; however, given the variable outcome measures and lack of more rigorous study designs, it is difficult to attribute specific scholarly concentration program outcomes to certain program features.

We used the Medical Education Research Study Quality Instrument [17] to evaluate the methodological rigour of the eleven studies included in our review. Mean scores were highest for data type, data analysis, and sampling, and lowest for study design, outcome, and study validity (Table 2). Most of the studies included in our review received maximum points for data type as they utilized objective data measurements rather than assessment by study participants. Studies also received high points for appropriateness of analysis to the study design and type of data; however, many of the studies included descriptive analyses only, which decreased overall data analysis domain scores. Response rates of studies that included a survey component were often greater than 75%, contributing to relatively high sampling domain scores. However, inclusion of data from only one institution reduced sampling domain scores for most studies. In addition, failure to report evaluation instrument validity, lack of patient and healthcare outcomes, and use of single group cross-sectional or single group post-test methodologies resulted in lower scores for study validity, outcome, and study design domains, respectively.

To gauge the relative quality of the studies included in our review, we compared domain and total scores with published studies that utilized the Medical Education Research Study Quality Instrument. Similar to our findings, Reed et al. scored 210 medical education studies and reported that mean domain scores were highest for data type, data analysis, and sampling, and were lowest for study validity and study design [17]. The average adjusted total score across the 210 studies was 9.95 [17]; the average adjusted total score of studies included in our review was 11.4. The overall reliability of the total scores was ‘almost perfect’ (0.92). Four items had ‘almost perfect’ reliability (0.83 to 1.00); one item had ‘substantial’ reliability (0.79); and one had ‘moderate’ reliability (0.56). The item ‘internal structure’ received a negative intraclass correlation coefficient estimate due to the limited range of scores, though there was very high agreement within papers (3/5 papers had 100% agreement). In addition, due to the limited range in scores between papers, the scale was not reliable for the items ‘relationships to other variables’ and ‘appropriateness of analysis’; however, the fraction of papers with 100% agreement among raters was 3/6 and 10/11 for these items, respectively. Hence, although intraclass correlation coefficient estimates were lower for certain items due to limited ranges in scores, the overall reliability among raters was high. For comparison, Reed et al. reported a Medical Education Research Study Quality Instrument item inter-rater reliability range from ‘substantial’ (0.72) to ‘almost perfect’ (0.98) [17].

Outcomes of educational programs are often difficult to evaluate and may not be detectable until several years after initiation [28]. For many educational interventions, randomization and controls are infeasible [11], the number of potential confounding factors may preclude generalizability to other settings, and lack of objective measures may limit study quality [12]. Despite these challenges, we advocate the use of rigorously designed, evidence-based research to evaluate scholarly concentration programs. As described by the Education Group for Guidelines on Evaluation, methods for program evaluation should be planned at the outset of the educational intervention and should be linked to the aims of the study [11]. Of the eleven papers included in our review, none reported evaluation methods that had been planned in advance of scholarly concentration program implementation; all of the studies either failed to specify the point at which evaluation methods were planned, or described evaluations that occurred in response to scholarly concentration program initiation [8, 13, 20–27, 29]. Furthermore, the aims of educational interventions should be reflected in both the aims of the research and in the methodology selected [11]. Only two papers in our review provided a weak rationale for the selected methodology and discussed aims of the research in the context of the intervention aims [8, 13]. In several of the studies, the methodology was poorly described, significantly detracting from the quality and reproducibility of the findings [20, 22, 29]. To achieve generalizability or reproducibility, the evaluation tool must be described in sufficient detail [11]; seven papers described the evaluation tool in sufficient detail for reproducibility [8, 13, 21, 23, 25, 26, 29]. Finally, given the challenges of randomized controlled trials in educational interventions, purposive sampling should be utilized to provide more informative results [11]. Only three papers in our review described purposive sampling methods [23–25].

For similar programs that are implemented in vastly different settings, credibility is improved and impact is more readily identified when a common outcome system is used [30]. Given the variability in scholarly concentration programs as well as the inherent challenges in scholarly concentration program evaluation, we propose the use of a conceptual framework as a foundation from which to identify and investigate important scholarly concentration program variables and outcome measures. Furthermore, the paucity of articles that met our inclusion criteria highlights the need for conceptual frameworks in evaluating scholarly concentration program characteristics. Logic models are a type of conceptual framework that can enhance understanding of the relationships between available resources, program activities, and desired changes or results [31]. Among the most common methods for generating logic models is the United Way approach [32], which defines four basic components: *inputs *(e. g., money, staff, time) are resources dedicated to or consumed by the program that are used to achieve program goals; *activities *are what the program does with inputs to fulfil its mission (e. g., strategies and techniques that comprise the program’s methodology); *outputs *are the direct products of program activities and are measured in terms of the volume of work accomplished (e. g., the number of participants served); and *outcomes *are benefits or changes for individuals or populations during or after participation in program activities [33].

**Fig. 2:**
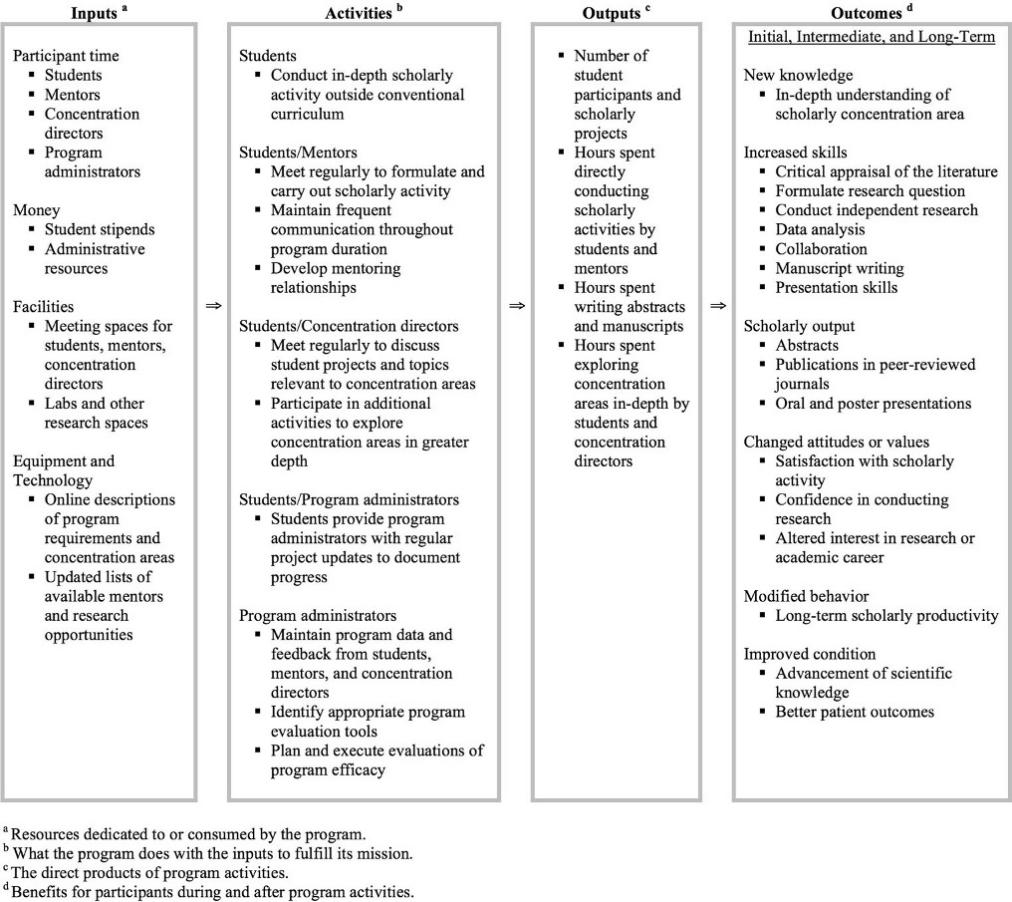
Scholarly concentration program outcome model

Drawing on the components outlined in the United Way model as well as common program measures included in papers from our review, we offer a logic model (Fig. 2) as a basic framework for program planners and educators to more rigorously evaluate scholarly concentration program characteristics and outcomes. Due to the limited number of papers in our review, we included components from each of the programs described, and did not consider Medical Education Research Study Quality Instrument scores in our data extraction (Supplementary file 2). Common themes and salient features among programs were then aggregated into a single conceptual framework. Our model highlights participant time, student stipends, administrative resources, and equipment and technology as *inputs*. Depending on the structure of the scholarly concentration program, evaluators may choose to assign different weights to each of these variables, or may include additional variables in order to more accurately characterize program resources. *Activities *in our model include student in-depth study outside the conventional curriculum, meetings among students, mentors, and concentration directors, and evaluations by program administrators to monitor student progress and program success. *Outputs *include number of participants and projects, and hours spent pursuing scholarly activities. *Outcomes *are either directly or indirectly impacted by *inputs, activities, *and *outputs, *and include new knowledge, increased skills, greater scholarly activity, modified behaviour and attitudes, and ultimately advancement of scientific knowledge and better patient outcomes. Each variable should be evaluated to determine the effects on other variables, and relationships among *inputs, activities, outputs, *and *outcomes *should be identified to more clearly understand the nature and function of individual programs. Importantly, a single conceptual framework is inherently limited in that only certain variables and their interrelatedness can be emphasized [34]; thus, in accordance with Schwab [35], we advocate the use of our logic model as merely a *basis* for evaluating scholarly concentration program characteristics and outcomes. Program planners and educators should ultimately develop multiple conceptual frameworks in order to view their program ‘through a succession of lenses’ [35].

Our study has several limitations. Though each rater received the same instructions and information on the Medical Education Research Study Quality Instrument scoring system, inter-rater reliability was less than ‘almost perfect’ for some of the domains. In addition, productivity in terms of student publications and presentations is only one measure of medical student scholarly activity and scholarly concentration program success. Other measures of scholarly concentration program success such as improved critical-thinking and analytical skills, career preparation, and student-faculty relationships are less tangible, albeit important indicators of scholarly concentration program efficacy. Furthermore, research productivity may not manifest as publications or presentations until several years after graduation, which may have led to the exclusion of certain articles from our study. Though no studies to date have examined whether students are more motivated by smaller-scale, shorter-term projects, we believe scholarly concentration programs should encourage students to complete research projects that yield tangible outcomes during their undergraduate medical education. Setting realistic goals and successfully achieving them are crucial to the ongoing motivation of medical students [36]; thus, shorter-term research projects that can be fully realized during the undergraduate medical years may serve as better motivators than longer-term projects or larger projects that lie outside students’ reach. Finally, we were unable to find any publically available data on scholarly concentration program costs or funding data for any medical student research programs. This data would help medical schools better evaluate the cost-benefit ratio of structured educational interventions such as scholarly concentration programs.

In summary, despite challenges inherent in medical education research, more rigorous, evidence-based studies that utilize conceptual frameworks are needed to determine how scholarly concentration programs and specific characteristics of scholarly concentration programs facilitate medical student research productivity. Comparative effectiveness research [37, 38] would also help define the benefits of scholarly concentration programs relative to other medical student research initiatives.

## Caption Electronic Supplementary Material

Supplementary file 1: Search strategies

Supplementary file 2: Data extraction worksheet for program outcome model
