# Supplementary material for: Scholarly concentration programs and medical student research productivity: a systematic review
Source: Perspect Med Educ. 2017 Mar 27;6(4):216–26. doi: 10.1007/s40037-017-0328-2 (PMC5542888; doi:10.1007/s40037-017-0328-2)
Supplement: Supplementary file 1 — Search strategies [file 40037_2017_328_MOESM1_ESM.docx]

**Supplementary file 1** Search strategies

**Pubmed** – Accessed March 11, 2016

("Students, Medical"[Mesh] OR "Schools, Medical"[Mesh] OR "Education, Medical"[Mesh:noexp] OR "education, medical, undergraduate"[mesh] OR “Medical students”[tiab] OR “medical schools”[tiab] OR “medical school”[tiab] OR “medical education”[tiab] OR “Medical students”[ot] OR “medical schools”[ot] OR “medical school”[ot] OR “medical education”[ot]) AND ("Research/education"[Majr] OR “research activity”[tiab] OR “research experience”[tiab] OR “research product”[tiab] OR “research products”[tiab] OR “scholarly concentration”[tiab] OR “scholarly concentrations”[tiab] OR “scholarly experience”[tiab] OR “scholarly experiences”[tiab] OR “scholarly activity”[tiab] OR “scholarly activities”[tiab] OR “scholarly program”[tiab] OR “scholarly programs”[tiab] OR “research activity”[ot] OR “research experience”[ot] OR “research product”[ot] OR “research products”[ot] OR “scholarly concentration”[ot] OR “scholarly concentrations”[ot] OR “scholarly experience”[ot] OR “scholarly experiences”[ot] OR “scholarly activity”[ot] OR “scholarly activities”[ot] OR “scholarly program”[ot] OR “scholarly programs”[ot])

**Embase** – Accessed 11 March 2016
('medical students' OR 'medical student' OR 'medical schools' OR 'medical school' OR 'medical education') AND ('scholarly concentrations' OR 'scholarly concentration' OR 'scholarly experience' OR 'scholarly experiences' OR 'scholarly activity' OR 'scholarly activities' OR 'scholarly program' OR 'scholarly programs' OR 'research productivity')

**Web of Science** – Accessed 11 March 2016

('medical students' OR 'medical student' OR 'medical schools' OR 'medical school' OR 'medical education') AND ('scholarly concentrations' OR 'scholarly concentration' OR 'scholarly experience' OR 'scholarly experiences' OR 'scholarly activity' OR 'scholarly activities' OR 'scholarly program' OR 'scholarly programs' OR 'research productivity')

**Teaching and Learning in Medicine** – Accessed 11 March 2016

('scholarly concentrations' OR 'scholarly concentration' OR 'scholarly experience' OR 'scholarly experiences' OR 'scholarly activity' OR 'scholarly activities' OR 'scholarly program' OR 'scholarly programs' OR 'research productivity' OR ‘research project’ OR ‘research projects’)

**Medical Education** – Accessed 11 March 2016

('scholarly concentrations' OR 'scholarly concentration' OR 'scholarly experience' OR 'scholarly experiences' OR 'scholarly activity' OR 'scholarly activities' OR 'scholarly program' OR 'scholarly programs' OR 'research productivity')

**Academic Medicine** – Accessed 11 March 2016

('scholarly concentrations' OR 'scholarly concentration' OR 'scholarly experience' OR 'scholarly experiences' OR 'scholarly activity' OR 'scholarly activities' OR 'scholarly program' OR 'scholarly programs' OR 'research productivity')
