# Supplementary material for: Scholarly concentration programs and medical student research productivity: a systematic review
Source: Perspect Med Educ. 2017 Mar 27;6(4):216–26. doi: 10.1007/s40037-017-0328-2 (PMC5542888; doi:10.1007/s40037-017-0328-2)
Supplement: Supplementary file 2 — Data extraction worksheet for program outcome model [file 40037_2017_328_MOESM2_ESM.docx]

**Supplementary file 2** Data extraction worksheet for program outcome model

| Studies included in review |  | Inputs | | |  | Activities |  | Outputs |  | Outcomes |  |
| --- | --- | --- | --- | --- | --- | --- | --- | --- | --- | --- | --- |
| Elwood et al. |  | | - Students - Mentors - Department staff |  | | - Student research project - Staff relationships and supervision - Seminars - Course in research methods |  |  |  | - Skills and attitudes - Publications and presentations - Utility for physician career and general education - Effect on practical and personal skills - Clinically-related skills, skills in doctor/patient relationships - Skills in research methods, interpersonal skills - Confidence - Influence on career choice |  |
| Gonzales et al. |  | | - Faculty mentors - Financial support for student research |  | | - Student research project - Research proposal - Research course - Coordination by predoctoral education office - Research agendas |  | - Number of students involved in research |  | - Publications and presentations |  |
| Smith et al. |  | | - Research program website and departmental websites - Poster board advertisements - Preceptor database - Faculty preceptors - Review committees |  | | - Research project program - Formal didactics - Small group sessions - Instruction in data handling, statistical analysis, data interpretation, writing a report - Identify a research question - Generate outline of proposed project - Teaching sessions customized to students’ projects - Develop instruments for data acquisition - Scientific or ethical review - Annual research symposium |  | - Contacts with faculty mentors |  | - Final written report - Publications and presentations at research symposium, local, national, international meetings - Research knowledge gained - Development of research skills (testing a hypothesis, data collection, data interpretation, data presentation) - Improved understanding of the research process - Skills (critically evaluating the literature, writing, library informatics searches, critical thinking, summarizing findings) - Understanding difficulties of research |  |
| Solomon et al. |  | | - Funding - Spaces for students to work (lab space, general clinical research centers, other sites) |  | | - Write a mini-proposal - Review of students’ proposals by faculty - Core lecture series - Ongoing research seminars and activities with preceptors’ departments - Questionnaire about students’ experience |  |  |  | - Final written report and presentation to classmates, or at regional or national meetings - Papers, abstracts, publication in peer-reviewed journals - Long-term effects (interest in academic career, career goals, career choice, thoughts about the value of research in a career) - Effects on conducting additional research after duration of the program, pursuing a second degree - Influence on obtaining residency of choice - Attitudes on the importance of biomedical research in improving patient care |  |
| Zier et al. |  | | - Financial support - Online resources to help students choose projects - Program support by the dean for medical education - Travel grants - Alumni association sponsors |  | | - Structured research programs - Medical student research day - Data collection on student publications - Survey of student experience - Meetings between associate dean and students to identify mentors |  | - Research awards and prizes |  | - Published papers - Level of student satisfaction with program - Effects on student interest in applying research to medical practice - Student perceptions on the level of project difficulty and learning - Effect of the program on interest in research |  |
| Dyrbye et al. |  | | - Faculty mentors - Funds for mentors |  | | - Required research experience - Review of proposals by research coordinating committee - Students identify mentor with help from faculty, deans, advisors - Write research proposal - Faculty supervise design of research projects, approve proposals, sponsor proposed projects, supervise projects, evaluate student performance |  |  |  | - Required research manuscript - Published research reports, published abstracts, presentations |  |
| Langhammer et al. |  | | - Office of Research and Sponsored Programs - Funding |  | | - Participation data collected - Student selection of a mentor - Students apply for funding - Workshops on grant writing, finding a mentor - Modules on clinical and translational research - Lectures introducing students to program and faculty - Program modification to meet student needs |  | - Number of supervised research activities each week |  | - Published manuscripts, presentations - Poster sessions - Required documents for program completion |  |

| Akman et al. |  | - Research mentors |  | - Student research program - Student congress - Scoring of student research reports and rating of student performance by mentors - Lectures - Research planning workshop - Presentation of research proposals - Annual collection of student feedback |  | - Hours of allocated student study time |  | - Research presentations at national or international meetings - Published papers and research reports not yet accepted for publication - Thoughts on project relation to future profession - Preparation of students for professional life - Skills (presentation, communication, statistics, literature searching, data gathering, data analysis, poster/oral presentations, writing a scientific paper) - Effect on self-confidence, decision to pursue a medical research career - Understanding causal relationships, development of scientific knowledge |  |
| --- | --- | --- | --- | --- | --- | --- | --- | --- | --- |
| Ogunyemi et al. |  | - Thesis committee (faculty and staff) - Research advisors, faculty mentors - Administrative support - Funding |  | - Primary care research thesis - Research orientation workshop - Thesis committee approval of research mentors and projects, progress monitoring, proposing award recipients, procuring resources for students and mentors, facilitating the publication process - Retreat for students, faculty, and staff at which program is reviewed and recommendations made for the following year - Forum during which students meet with potential faculty mentors - Students conduct research project, adhere to timeline, and submit assignments - Workshops on Medline searches, research design, IRB process, data collection, statistical software |  |  |  | - Publications and presentations at an annual research day - Program effects on knowledge of the research process, improving competitiveness for residency, effects on student interest in research - Student satisfaction from accomplishing a goal, - Effect on encouraging students to become leaders in academic endeavors |  |
| Areephanthu et al. |  | - Faculty mentors - Stipends for students |  | - Introductory course - Students identify mentor, develop hypothesis-driven proposal, apply for program - Core seminars and research lecture series - Participation in annual student research conference - Students engage in self-reflection and are evaluated by mentors - Student research project |  |  |  | - Published papers - Effect on student understanding of research design and execution - Effects on board examination performance and overall academic performance |  |
| George et al. |  | - Stipends for students - Faculty mentors - Interdisciplinary faculty |  | - Students write and submit application - Discussions and didactic sessions |  |  |  | - Submission of scholarly products - Number of publications in peer-reviewed journals - Effects on career choice and residency placement |  |
